# Lactylation in sepsis-associated acute kidney injury: regulatory mechanisms and therapeutic prospects

**DOI:** 10.3389/fmolb.2026.1899336

**Published:** 2026-08-19

**Authors:** Jiahui Zheng, Boyang Zheng, Qun Liang

**Affiliations:** 1 Graduate School, Heilongjiang University of Chinese Medicine, Harbin, China; 2 Department of Critical Care Medicine, First Affiliated Hospital of Heilongjiang University of Chinese Medicine, Harbin, China

**Keywords:** AKI-to-CKD transition, immune regulation, lactate metabolism, lactylation, lysine lactylation, mitochondrial dysfunction, sepsis-associated acute kidney injury

## Abstract

**Introduction:**

Sepsis-associated acute kidney injury (SA-AKI) is a prevalent, life-threatening sepsis complication with high mortality, prolonged organ support dependence, and scarce targeted therapies. Beyond being an anaerobic glycolysis byproduct, lactate serves as a critical circulating carbon source, mitochondrial fuel, redox regulator, signaling molecule, and lysine lactylation (Kla) substrate. Its multifaceted functions are vital to SA-AKI pathogenesis, which involves systemic lactate overload, impaired lactate clearance, renal metabolic reprogramming, and abnormal immune activation.

**Methods:**

This review synthesizes up-to-date evidence to systematically elucidate lactate and Kla mechanisms in SA-AKI. We hierarchically integrate findings from systemic sepsis metabolism and renal tubular lactate handling to cell-specific Kla modifications, aiming to clarify their distinct roles in SA-AKI progression.

**Results:**

Specific Kla sites (H3K18la, Fis1 K20la, LDHB K156la, Ezrin K263la, HMGB1 lactylation, ALDH2 K68la) mediate SA-AKI pathologies including mitochondrial dysfunction, tubular death, endothelial injury, and cGAS-STING/NLRP3-neutrophil extracellular trap activation. Lactate accumulation, acidosis, transport, oxidation, metabolic routing, and Kla are mechanistically distinct rather than uniformly harmful. Lactate/pyruvate metabolism exerts context-dependent injurious or adaptive effects across kidney disease models, modulated by cell type, injury phase, and metabolic reserve.

**Discussion:**

Lactate- and Kla-targeted strategies are promising for SA-AKI treatment yet require rigorous clinical validation. Blood lactate level and clearance are reliable clinical prognostic biomarkers, whereas Kla signatures remain investigational. Balanced understanding of lactate-Kla biology will refine precision diagnostic and therapeutic strategies for SA-AKI, advancing translational clinical application.

## 1 Introduction

Sepsis, defined as life-threatening organ dysfunction resulting from a dysregulated host response to infection (Singer et al., 2016), It remains a major global health burden and is accompanied by complex metabolic, inflammatory, endothelial, and mitochondrial disturbances (Gray et al., 2025; Peerapornratana et al., 2019; Zarbock et al., 2023). SA-AKI is one of its most common organ complications, affecting a substantial proportion of critically ill septic patients and substantially increasing short-term and long-term mortality (Zarbock et al., 2023; Poston and Koyner, 2019). The pathogenesis of SA-AKI cannot be explained by renal hypoperfusion alone. Current models emphasize microcirculatory dysfunction, tubular epithelial cell stress, mitochondrial injury, endothelial activation, regulated cell death, immunometabolic reprogramming, and maladaptive repair (Peerapornratana et al., 2019; Zarbock et al., 2023; Gómez and Kellum, 2019; Liu A-B. et al., 2024; Liu C. et al., 2024; Pickkers et al., 2021). Lactate has historically been used as a clinical marker of tissue hypoperfusion and disease severity. This interpretation remains clinically useful, particularly when lactate clearance is considered alongside organ dysfunction and hemodynamic status (Ryoo et al., 2018). Yet lactate biology is broader than lactic acidosis. Lactate is continuously produced and consumed under aerobic conditions, moves between organs and cell compartments through lactate-shuttle mechanisms, contributes carbon to the tricarboxylic acid (TCA) cycle, and participates in signaling and immune regulation (Brooks, 2018; Rabinowitz and Enerbäck, 2020; Certo et al., 2022; Li et al., 2022; Hui et al., 2017; Brooks, 2009). The discovery of histone lactylation in 2019 further linked lactate availability to chromatin regulation and post-translational modification (Zhang et al., 2019; Liberti and Locasale, 2020).

**FIGURE 1:**
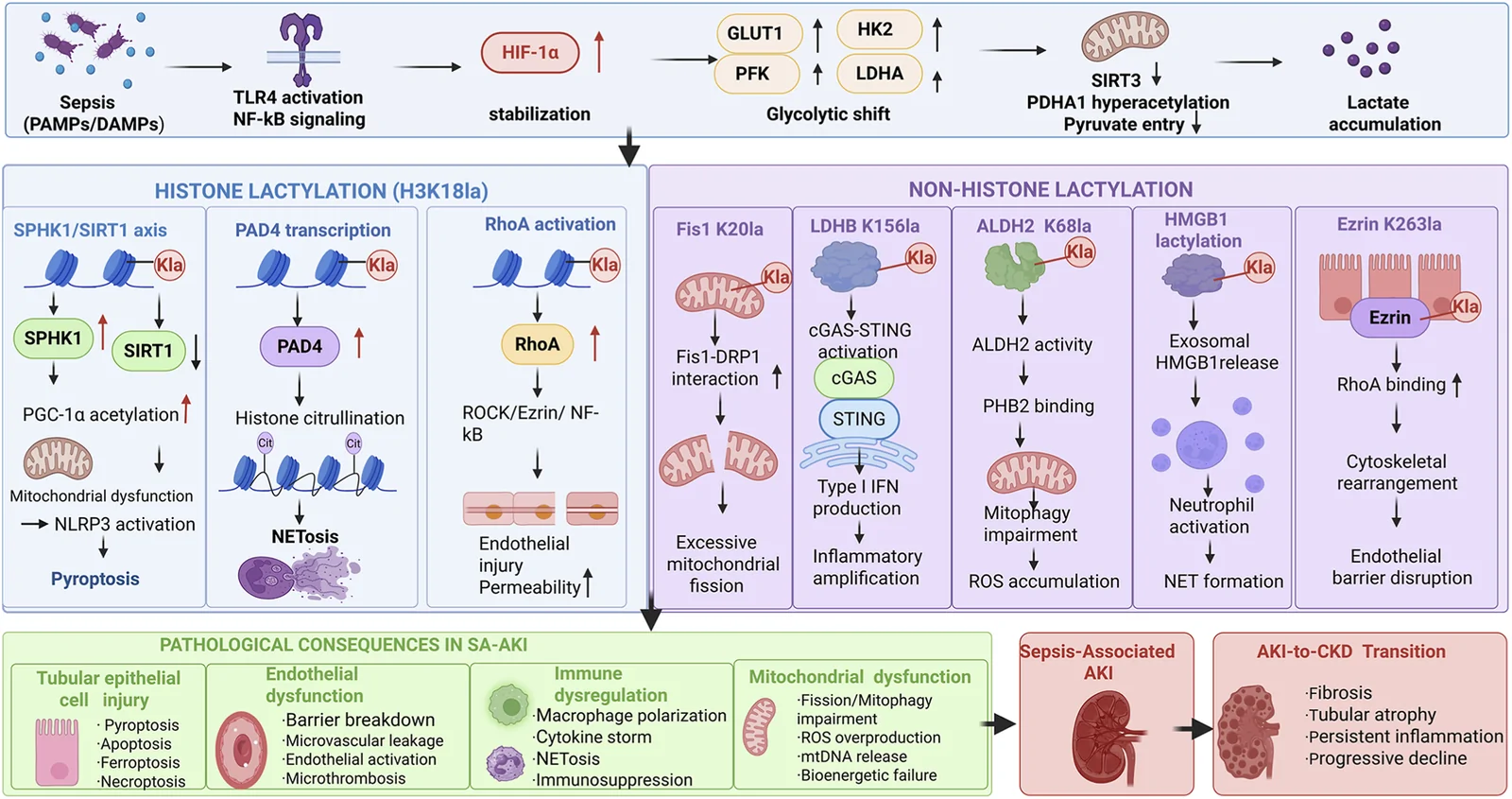
Lactate Metabolic Reprogramming and Lactylation-related mechanisms in SA-AKI.

In SA-AKI, lactate and lactylation have attracted attention because direct and emerging studies have reported increased renal lactate burden, enhanced protein lactylation, and site-specific histone or non-histone lactylation events in tubular epithelial cells, endothelial cells, macrophages, and neutrophil-associated pathways (An et al., 2023; Zhang et al., 2025; Huang et al., 2026; Qiao et al., 2024; Luo et al., 2026; Luo et al., 2025; Zhu et al., 2024; Wei et al., 2025; Huang et al., 2025). At the same time, several mechanisms cited in this field are derived from systemic sepsis biology, ischemia-reperfusion injury, cisplatin or rhabdomyolysis-induced AKI, diabetic kidney disease, CKD, acute lung injury, or general lactylation biology. This review therefore distinguishes direct SA-AKI evidence from inferred mechanisms and reorganizes the literature around biological outcomes and cell types rather than isolated signaling pathways. Table 1 and Figure 1.

**TABLE 1:** Evidence map for lactate metabolism and lactylation in SA-AKI and related kidney injury models.

Model/source	Evidence level	Cell/tissue focus	Lactate/lactylation feature	Biological outcome	Translational interpretation	References
MIMIC-IV cohort + CLP model	Direct clinical association + direct animal SA-AKI	Systemic lactate; kidney tissue	Blood lactate dynamics; renal protein lactylation	Higher SA-AKI risk; kidney-selective lactylation signal	Supports risk stratification; lactylation biomarker still investigational	Zhang et al. (2025)
CLP mice + HK-2 cells	Direct experimental SA-AKI	Tubular epithelial cells	SIRT3/PDHA1-driven lactate accumulation; Fis1 K20la	Excessive mitochondrial fission; apoptosis	Supports context-specific metabolic intervention	An et al. (2023)
Septic kidney tissue + tubular cells	Direct experimental SA-AKI	Tubular epithelial cells	H3K18la at SPHK1 promoter; SIRT1 feedback	Mitochondrial dysfunction; NLRP3-mediated pyroptosis	SIRT1/SPHK1/p300 axis is a candidate target	Huang et al. (2026)
CLP mice + endothelial cells	Direct experimental SA-AKI	Renal endothelial cells	H3K18la/RhoA and Ezrin K263la	Cytoskeletal remodeling; vascular leakage	ROCK/Ezrin axis may protect barrier function	Qiao et al. (2024)
Septic AKI model	Direct experimental SA-AKI	Neutrophil-related renal inflammation	H3K18la-mediated PAD4 upregulation	NETosis; microvascular inflammation	PAD4/NETosis inhibition is a potential downstream approach	Huang et al. (2025)
Septic tubular model	Direct experimental SA-AKI	Tubular epithelial cells	LDHB K156la	cGAS-STING/NLRP3 activation; pyroptosis	Innate immune signaling is a downstream target	Luo et al. (2026)
IRI and sepsis-induced AKI models	Direct AKI evidence; partly SA-AKI-related	Tubular mitochondria	ALDH2 K68la	Impaired aldehyde detoxification and PHB2-mediated mitophagy	Mitochondrial quality control target; SA-AKI validation needed	Li et al. (2025)
Macrophage/septic AKI models	Direct experimental SA-AKI	Macrophage-neutrophil crosstalk	HMGB1 lactylation and exosomal release	NET formation; thrombosis; inflammation	HMGB1/NETs axis is a plausible target	Zhu et al. (2024); Wei et al. (2025)
SA-AKI transcriptomic dataset	Computational/hypothesis-generating	Proximal tubule/loop of Henle; immune cells	LRG signatures; PECR and TP53I3	Immune heterogeneity and molecular subtypes	Requires prospective and experimental validation	Jiang et al. (2025)
Diabetic nephropathy/renal fibrosis models	Indirect renal disease evidence	Tubular/fibrotic compartments	Lactate/AARS1/H3K18la/LDHA; ACSL4-related pathways	Ferroptosis and fibrosis	Relevant to AKI-to-CKD hypothesis but not direct SA-AKI proof	Hu et al. (2025), Xiang et al. (2025)
AKI-to-CKD model	Direct AKI-to-CKD evidence; not sepsis-specific	Tubular mitochondria/inflammasome	Citrate synthase lactylation	NLRP3 activation and chronic transition	Supports lactylation-memory hypothesis; septic validation needed	Yu et al. (2025)
Cisplatin AKI; rhabdomyolysis AKI	Indirect AKI counter-evidence	Proximal tubules	LDHA deletion; MPC disruption	LDHA loss worsens AKI; MPC disruption can protect via redox adaptation	Warns against universal LDHA inhibition or pyruvate-oxidation assumptions	Lu et al. (2026), Rauckhorst et al. (2023), Rauckhorst et al. (2024)

Evidence levels distinguish direct SA-AKI, studies from indirect AKI, CKD, diabetic nephropathy, fibrosis, systemic sepsis, or computational evidence.

## 2 Systemic lactate metabolism in sepsis: homeostatic and maladaptive contexts

### 2.1 Lactate production, clearance, transport, and lactic acidosis are distinct processes

During sepsis, circulating lactate can rise because of accelerated glycolysis, adrenergic stimulation, inflammatory activation, mitochondrial stress, hepatic dysfunction, renal clearance changes, impaired tissue extraction, or combinations of these mechanisms. Therefore, hyperlactatemia should not be equated automatically with anaerobic metabolism or tissue hypoxia. Lactic acidosis is also not identical to lactate accumulation: the measured lactate anion reflects a metabolic flux state, whereas blood pH is shaped by buffering, ventilation, renal handling, strong-ion balance, and coexisting acid-base disorders (Brooks, 2018; Rabinowitz and Enerbäck, 2020; Certo et al., 2022; Li et al., 2022).

Lactate transport and utilization add another level of complexity. Monocarboxylate transporters (MCTs), lactate dehydrogenase isoforms, GPR81/HCAR1 signaling, and organ-to-organ exchange determine whether lactate is exported, oxidized, converted to pyruvate, used for gluconeogenesis, or retained as a substrate for lactylation (Brooks, 2018; Certo et al., 2022; Li et al., 2022; Brooks, 2009). In septic patients, blood lactate and lactate clearance remain clinically relevant markers of risk and resuscitation response (Ryoo et al., 2018; Zhang et al., 2025). However, these circulating indices cannot by themselves identify intracellular lactate routing, renal cell lactylation burden, or site-specific protein modifications.

### 2.2 Adaptive lactate functions and the limits of a purely pathogenic model

A central revision of this manuscript is to avoid a one-sided interpretation of lactate as exclusively deleterious. Experimental isotope-tracing studies have shown that circulating lactate can feed the TCA cycle and may function as a major carbon source for energy metabolism (Hui et al., 2017). Lactate-shuttle theory further emphasizes lactate as an oxidative substrate, gluconeogenic precursor, and signaling molecule (Brooks, 2009). More recently, lactate was reported to activate the mitochondrial electron transport chain independently of its metabolism, indicating that lactate may also support bioenergetic adaptation under certain conditions (Cai et al., 2023).

Immunologically, lactate may suppress excessive inflammation through GPR81-dependent and GPR81-independent mechanisms, including inhibition of inflammasome activation, reduced macrophage pro-inflammatory signaling, and promotion of anti-inflammatory or reparative macrophage phenotypes (Hoque et al., 2014; Er et al., 2016; Yang et al., 2020; Zhang et al., 2021). These effects do not negate the association between persistent hyperlactatemia and poor outcomes in sepsis. Rather, they indicate that the biological meaning of lactate depends on timing, concentration, tissue compartment, receptor/transporter expression, redox state, and cellular metabolic flexibility. In SA-AKI, the key question is therefore not whether lactate is “good” or “bad,” but when lactate flux supports adaptation and when sustained lactate availability drives maladaptive lactylation, inflammatory amplification, or mitochondrial injury.

## 3 Renal tubular lactate handling and metabolic routing in sepsis

### 3.1 Tubular metabolic reprogramming and lactate generation

Renal tubular epithelial cells are highly energy-demanding and depend heavily on mitochondrial oxidative metabolism. In sepsis, pathogen-associated molecular patterns such as lipopolysaccharide activate TLR4-mediated NF-κB and PI3K/Akt signaling, stabilize HIF-1α, and increase expression of glycolytic enzymes including HK2, PFK, and LDHA (Tannahill et al., 2013; Cheng et al., 2014; Kierans and Taylor, 2021). HIF-1α can also induce PDK1, thereby inhibiting PDHA1 and decreasing pyruvate entry into the TCA cycle. This shift supports short-term glycolytic ATP production but may increase lactate availability in a kidney already exposed to systemic hyperlactatemia, inflammatory cytokines, endothelial dysfunction, and mitochondrial stress (Liu A-B. et al., 2024; Liu C. et al., 2024).

An et al. reported an additional SA-AKI mechanism in which SIRT3 downregulation promotes PDHA1 K385 hyperacetylation, reducing pyruvate oxidation and increasing lactate production in tubular epithelial cells. In their model, lactate accumulation was linked to Fis1 K20 lactylation, excessive mitochondrial fission, and tubular injury (An et al., 2023). This provides direct SA-AKI evidence that tubular lactate overproduction can be pathogenic under specific conditions. Still, the result should be interpreted within the septic context and not generalized to all AKI models or all renal cell types.

### 3.2 Pyruvate-lactate routing: Injury or adaptation

Interventions in lactate/pyruvate metabolism may have divergent effects. Evidence outside sepsis indicates that proximal tubular LDHA can support metabolic flexibility and injury tolerance: proximal tubule-specific LDHA deletion worsened cisplatin-induced AKI rather than protecting it (Lu et al., 2026). Similarly, reduced mitochondrial pyruvate uptake through tubular mitochondrial pyruvate carrier (MPC) disruption protected against rhabdomyolysis-induced AKI by inducing redox adaptations and improving survival in the updated peer-reviewed study (Rauckhorst et al., 2023; Rauckhorst et al., 2024). These findings require caution when translating LDH inhibition, PDK inhibition, or pyruvate-routing strategies across AKI etiologies.

A working model is that glycolysis-derived lactate may become injurious when it sustains pathological lactylation, excessive mitochondrial fission, inflammatory signaling, or endothelial leakage in septic kidneys. Conversely, altered pyruvate/lactate routing may be adaptive when it preserves NAD^+^/NADH balance, supports glycolytic survival, fuels neighboring cells, limits mitochondrial overload, or triggers antioxidant defenses. The distinction is likely cell-type- and context-dependent. Proximal tubules, endothelial cells, macrophages, neutrophils, and fibroblasts differ in mitochondrial density, lactate transporter expression, LDH isoform balance, redox state, and lactylation enzyme activity. Future SA-AKI studies should therefore measure lactate production, transport, oxidation, acid-base effects, and lactylation separately rather than treating them as a single pathogenic axis.

## 4 Molecular basis of lysine lactylation

### 4.1 Biochemical fundamentals

Lysine lactylation is a post-translational modification in which lactate-derived lactyl groups are covalently attached to lysine residues. Lactylation occurs on histone and non-histone proteins (Zhang et al., 2019; Liberti and Locasale, 2020). Histone lactylation can influence gene transcription by changing chromatin states or transcription-factor accessibility, whereas non-histone lactylation can alter enzymatic activity, protein stability, subcellular localization, protein-protein interactions, or organelle function (Zhang et al., 2019; Liberti and Locasale, 2020; Moreno-Yruela et al., 2022). In renal disease, lactylation belongs to a broader network of acyl modifications that includes acetylation, crotonylation, succinylation, malonylation, and other lysine modifications (Sabari et al., 2017; Liu et al., 2023).

Mechanistic separation is essential. Lactate accumulation increases substrate availability, but it does not automatically prove site-specific lactylation or causality. Lactic acidosis may independently influence enzyme activity, mitochondrial function, and inflammatory responses. Lactate transport determines intracellular substrate access. Lactate oxidation and pyruvate routing shape redox and TCA-cycle flux. Lysine lactylation, in contrast, is a protein-level modification requiring modification-specific detection and functional validation. For this reason, mechanistic claims in this review are graded by model and evidence level in the tables.

### 4.2 Writers, erasers, and readers

The best-characterized lactylation writer is p300/CBP, which can use lactyl-CoA to catalyze histone lactylation (Zhang et al., 2019). AARS1 and AARS2 were later reported to sense L-lactate and function as lysine lactyltransferases, expanding the enzymatic framework of lactylation biology (Li et al., 2024). Class I HDACs and sirtuins have been implicated as delactylases; in SA-AKI, SIRT1 has been experimentally linked to H3K18la removal and negative feedback regulation in the SPHK1/SIRT1 axis (Huang et al., 2026; Moreno-Yruela et al., 2022). Fewer lactylation readers have been identified, and the reader layer remains a major knowledge gap (Zhang et al., 2026; Xu et al., 2025).

## 5 Cell-specific and outcome-oriented roles of lactylation in SA-AKI

### 5.1 Tubular epithelial mitochondrial dysfunction, pyroptosis, and apoptosis

Mitochondrial injury is a central biological outcome of lactylation-related mechanisms in SA-AKI. Huang et al. reported that H3K18la is enriched at the SPHK1 promoter in septic kidney tissue, promoting SPHK1 transcription through a p300-dependent mechanism (Huang et al., 2026). SPHK1 then phosphorylates SIRT1 at Ser47, promoting ubiquitin-proteasome degradation of SIRT1. Reduced SIRT1 activity leads to PGC-1α hyperacetylation and impaired mitochondrial biogenesis, followed by mitochondrial ROS accumulation and NLRP3 inflammasome activation. SIRT1 also functions as an H3K18la delactylase in this setting, suggesting an unstable feedback system in which persistent lactylation may overcome negative regulation.

Non-histone lactylation provides a complementary mitochondrial mechanism. Fis1 K20 lactylation strengthens Fis1-DRP1 interaction, promotes DRP1 recruitment to mitochondria, and drives excessive mitochondrial fission in septic tubular cells (An et al., 2023). LDHB K156 lactylation links metabolic reprogramming to cGAS-STING and NLRP3 inflammasome activation, thereby connecting mitochondrial stress, innate immune sensing, and tubular pyroptosis (Luo et al., 2026). ALDH2 K68 lactylation, reported in ischemia-reperfusion and sepsis-induced AKI models, impairs aldehyde detoxification and disrupts PHB2-mediated mitophagy, which may exacerbate mitochondrial oxidative damage (Li et al., 2025). Together, these findings support mitochondrial dysfunction as a major biological outcome, but the degree of direct SA-AKI evidence varies among targets (Table 2).

**TABLE 2:** Regulators of lactate biology and lysine lactylation relevant to SA-AKI.

Category	Molecule/process	Biological function	Potential role in SA-AKI	Evidence/model	Therapeutic relevance	References
Systemic metabolism	Lactate production and clearance	Reflects glycolytic flux, organ exchange, tissue extraction, hepatic/renal clearance	Risk marker; does not prove intracellular lactylation	Clinical sepsis association	Useful for monitoring; not a direct lactylation biomarker	Ryoo et al. (2018); Zhang et al. (2025)
Transport/sensing	MCTs and GPR81/HCAR1	Lactate transport and receptor-mediated signaling	May influence endothelial, tubular, and immune responses	Renal and immune models	Transport/receptor targeting requires cell specificity	Certo et al. (2022), Hoque et al. (2014), Er et al. (2016), Yang et al. (2020), Jones et al. (2020), Grochowalska et al. (2022)
Metabolic routing	LDHA/LDHB/PDHA1/MPC	Controls pyruvate-lactate conversion and mitochondrial pyruvate entry	Can drive or buffer injury depending on context	Direct SA-AKI plus indirect AKI evidence	Avoid universal LDHA inhibition assumptions	An et al. (2023), Luo et al. (2026), Lu et al. (2026), Rauckhorst et al. (2023), Rauckhorst et al. (2024)
Writer	p300/CBP	Histone and non-histone lactyltransferase activity	Promotes H3K18la-linked transcription in several SA-AKI pathways	Direct experimental SA-AKI and general lactylation biology	Potential target; specificity and safety unclear	Zhang et al. (2019), Huang et al. (2026), Qiao et al. (2024), Luo et al. (2025), Huang et al. (2025)
Writer	AARS1/AARS2	L-lactate-sensing lysine lactyltransferases	Potential contributor to lactylation landscape	General lactylation and renal disease evidence	Emerging target; SA-AKI role undefined	Li et al. (2024), Xu et al. (2025)
Eraser	SIRT1	NAD + -dependent deacetylase/delactylase	Removes H3K18la and restrains SPHK1/SIRT1 feedback	Direct experimental SA-AKI	SIRT1 activation/NAD + may be protective	Huang et al. (2026)
Eraser/metabolic regulator	SIRT3	Mitochondrial deacetylase regulating PDHA1	SIRT3 loss promotes lactate overproduction and Fis1 lactylation	Direct experimental SA-AKI	Supports mitochondrial metabolic rescue	An et al. (2023), Yang et al. (2025), Jin et al. (2024)
Eraser	HDAC1–3	Histone lysine delactylases	May regulate inflammatory chromatin states	General lactylation biology	SA-AKI specificity remains unclear	Moreno-Yruela et al. (2022)
Reader	TRIM33 and putative readers	Recognition of lactylated lysine marks	Reader layer in renal lactylation is poorly defined	General/indirect evidence	Major knowledge gap for precision targeting	Zhang et al. (2026), Xu et al. (2025)
Downstream signaling	NLRP3/cGAS-STING/ROCK/NETosis	Inflammasome, innate immune, cytoskeletal and neutrophil pathways	Execute cellular injury downstream of lactylation events	Direct and indirect SA-AKI evidence	May be more targetable than lactylation itself	Huang et al. (2026), Qiao et al. (2024), Luo et al. (2026), Zhu et al., 2024; Wei et al. (2025), Huang et al. (2025), N et al., 2025; Fu et al. (2025)

The table separates lactate metabolism/transport from lysine lactylation enzymes and downstream effector pathways.

### 5.2 Endothelial barrier dysfunction and microvascular leakage

Renal endothelial injury is another outcome-based mechanism. Qiao et al. showed that H3K18la is increased in septic renal tissues and enriched at the RhoA promoter, promoting RhoA transcription and ROCK/Ezrin signaling (Qiao et al., 2024) The same study identified Ezrin K263 lactylation, which enhanced Ezrin-RhoA interaction and promoted cytoskeletal reorganization, junctional disruption, and vascular leakage. ROCK inhibition attenuated this endothelial injury in the experimental model. These data place endothelial lactylation at the interface of metabolic stress, cytoskeletal remodeling, and microcirculatory dysfunction, a key component of SA-AKI pathophysiology.

### 5.3 Immune-cell crosstalk, macrophage polarization, and NETosis

Immune-cell mechanisms should be interpreted as systemic and renal microenvironmental processes rather than exclusively kidney-intrinsic events. Macrophages and neutrophils are exposed to systemic lactate, tissue lactate gradients, circulating danger signals, and endothelial activation. Lactate-induced HMGB1 lactylation in macrophages promotes exosomal HMGB1 release and neutrophil extracellular trap (NET) formation, thereby aggravating renal inflammation and microvascular thrombosis in SA-AKI models (Zhu et al., 2024; Wei et al., 2025). In parallel, H3K18la-mediated PAD4 upregulation promotes NETosis in septic AKI, further linking lactylation to neutrophil-driven microvascular injury (Huang et al., 2025). NETs are increasingly recognized as contributors to microvascular damage, inflammation, and fibrosis in kidney disease (N et al., 2025; Fu et al., 2025).

Macrophage polarization illustrates the adaptive-maladaptive duality of lactate. Lactate and lactylation can induce reparative or M2-associated transcriptional programs, as shown in the original histone lactylation study and in additional macrophage studies (Zhang et al., 2019; Hoque et al., 2014; Er et al., 2016; Yang et al., 2020; Zhang et al., 2021). However, in SA-AKI, similar immunometabolic remodeling may become pathogenic if it amplifies HMGB1 release, NET formation, immune paralysis, or maladaptive repair (Zhu et al., 2024; Wei et al., 2025; Wen et al., 2021; Sun et al., 2026; Shu et al., 2025). Bioinformatic analyses have identified lactylation-related gene signatures and immune-infiltration patterns in SA-AKI datasets, including increased macrophage and neutrophil signals and possible molecular subtypes (Jiang et al., 2025). These findings are hypothesis-generating and require prospective validation and experimental confirmation.

### 5.4 Lactylation and the AKI-to-CKD transition

Incomplete repair after AKI can progress to tubulointerstitial fibrosis and CKD (Fiorentino et al., 2018). For SA-AKI, direct longitudinal evidence linking specific lactylation marks to AKI-to-CKD transition remains limited. Nevertheless, several renal disease studies suggest plausible mechanisms. In diabetic nephropathy, a lactate/AARS1/H3K18la/LDHA positive feedback loop may support persistent lactate accumulation and ferroptosis-related injury (Hu et al., 2025). Fibrosis studies implicate lactylation in TGFβ-linked signaling and ferroptosis (Xiang et al., 2025). More directly, protein lactylation of citrate synthase has been reported to promote AKI-to-CKD transition by activating the NLRP3 inflammasome (Yu et al., 2025).

**FIGURE 2:**
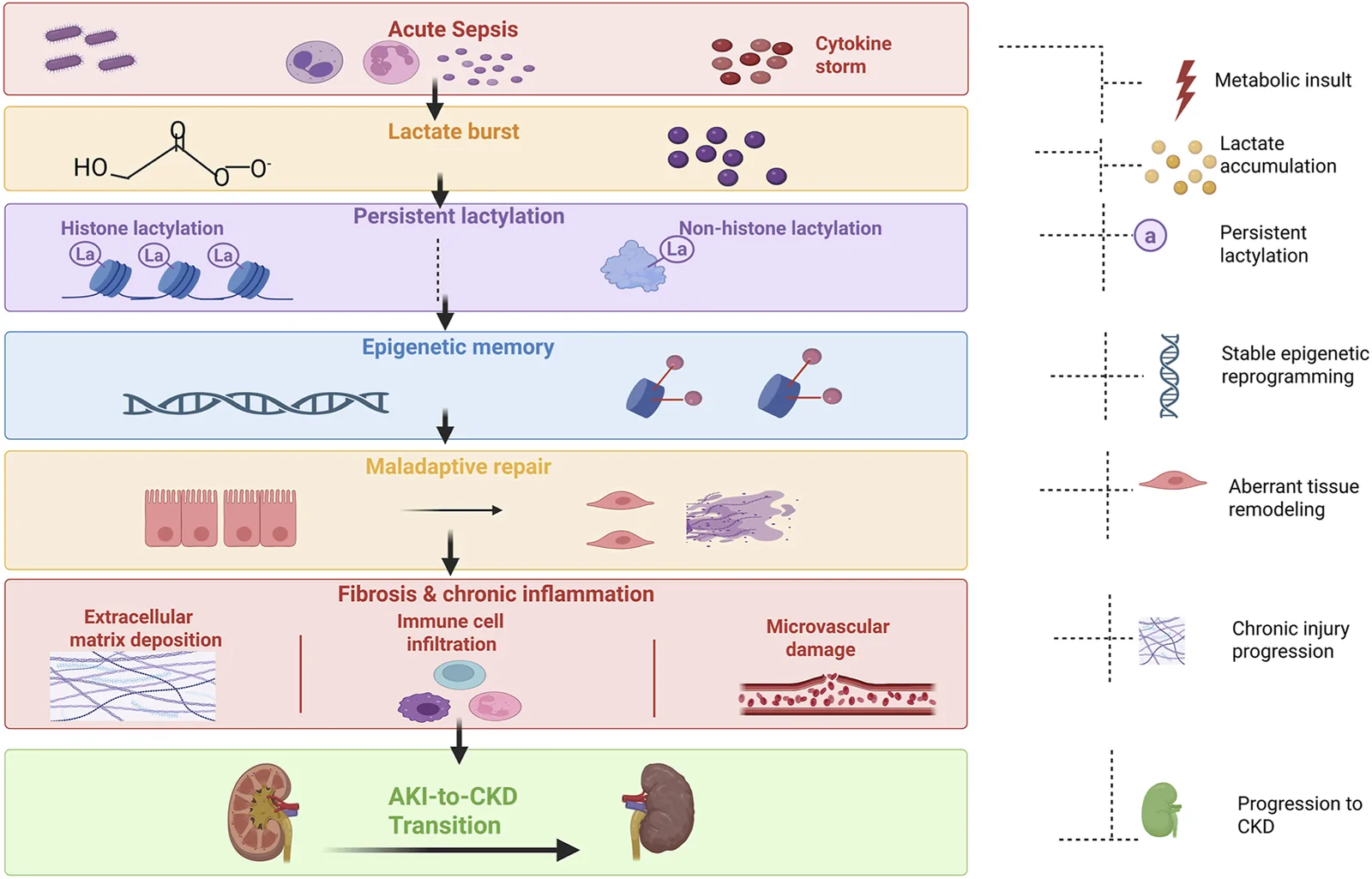
Potential role of persistent lactylation in the AKI-to-CKD transition following sepsis. Evidence note: this figure illustrates a hypothesis. Direct septic longitudinal validation is not yet available; evidence is mainly derived from AKI-to-CKD, CKD, and renal fibrosis models.

These findings justify discussing lactylation as a potential mediator of metabolic epigenetic memory, but they do not prove that septic lactylation signatures independently predict CKD progression. A cautious formulation is therefore warranted: lactylation may contribute to maladaptive repair when persistent histone or non-histone marks maintain mitochondrial dysfunction, inflammatory signaling, or profibrotic transcription after the septic insult resolves (Yu et al., 2025; Kwoun et al., 2025; Zhu et al., 2025). Prospective SA-AKI cohorts with serial kidney function, urine or blood biomarkers, and cell-type-resolved lactylomics will be needed to test this hypothesis (Figure 2).

## 6 Therapeutic implications and limitations

### 6.1 Targeting lactate metabolism and transport with caution

Reducing excessive lactate production may attenuate pathological lactylation in some SA-AKI models. DCA, a PDK inhibitor, restored pyruvate oxidation, reduced renal lactate burden, decreased Fis1 K20la, and improved renal injury in septic mice (An et al., 2023). LDH inhibitors such as GSK2837808A and oxamate reduced lactate-associated injury markers in selected experimental settings (An et al., 2023; Zhu et al., 2024). However, LDH or LDHA inhibition should not be presented as a universally protective strategy. Proximal tubule LDHA deletion worsened cisplatin-induced AKI, indicating that tubular LDHA may preserve metabolic flexibility and injury tolerance in some contexts (Lu et al., 2026).

These observations argue for context-sensitive therapy. In septic kidneys with sustained lactate overproduction and pathological lactylation, limiting upstream lactate production may be beneficial. In other AKI models, preserving lactate production or reducing mitochondrial pyruvate uptake may be protective by sustaining glycolysis, NAD^+^ regeneration, or antioxidant adaptation (Lu et al., 2026; Rauckhorst et al., 2023; Rauckhorst et al., 2024). Future drug development should therefore identify the target cell type, injury stage, and dominant metabolic defect before inhibiting lactate production or transport.

### 6.2 Targeting lactylation writers, erasers, and downstream effectors

Directly targeting lactylation enzymes may offer greater specificity than globally suppressing lactate metabolism. NAD^+^ supplementation and SIRT1 activation may enhance delactylation and mitochondrial homeostasis in the H3K18la/SPHK1/SIRT1 axis, while SPHK1 inhibition may disrupt a pathogenic feedback loop in experimental SA-AKI (Huang et al., 2026). p300/CBP inhibition is mechanistically attractive because p300 contributes to histone lactylation, but specificity and safety remain major concerns (Zhang et al., 2019; Huang et al., 2026; Qiao et al., 2024; Luo et al., 2025; Huang et al., 2025).

Downstream pathway inhibition is another practical strategy. Mdivi-1 targets excessive mitochondrial fission downstream of Fis1 lactylation (An et al., 2023). Y-27632 targets the RhoA/ROCK/Ezrin axis and may protect endothelial barrier integrity (Qiao et al., 2024). MCC950 and H-151 target NLRP3 inflammasome and cGAS-STING pathways downstream of lactylation-associated mitochondrial and innate immune activation (Huang et al., 2026; Luo et al., 2026). Glycyrrhizin and HMGB1 silencing attenuate HMGB1-related NET formation in lactate-induced AKI models (Zhu et al., 2024). These approaches are not lactylation-specific and should be described as downstream modulators rather than direct anti-lactylation therapies.

### 6.3 Natural compounds and traditional Chinese medicine-derived strategies

Several natural compounds may intersect with lactate-lactylation biology. Curcumin reduced p300 expression, protein lactylation, inflammation, and kidney injury in a CLP-induced SA-AKI model (Luo et al., 2025). Salvianolic acid C reduced H3K18la-associated inflammatory responses through a PGC1α-gluconeogenesis-histone lactylation axis in SA-AKI (Hong et al., 2026). EGCG has been reported to inhibit p300-related lactylation in diabetic nephropathy, but this remains indirect evidence for SA-AKI (Hong et al., 2026; Cheng and Guo, 2025). Such compounds may be useful as mechanistic probes or adjunctive leads, yet their bioavailability, target specificity, dosing windows, and safety in septic patients require rigorous evaluation.

### 6.4 Biomarkers, patient stratification, and translational barriers

Biomarker interpretation should remain cautious. Blood lactate and lactate clearance are clinically relevant for sepsis risk assessment and dynamic monitoring (Ryoo et al., 2018; Zhang et al., 2025). By contrast, protein lactylation signatures are not yet validated diagnostic, prognostic, or AKI-to-CKD prediction biomarkers for SA-AKI. Their clinical translation requires standardized assays, pre-analytical control, tissue or biofluid source selection, prospective cohorts, and independent validation.

**FIGURE 3:**
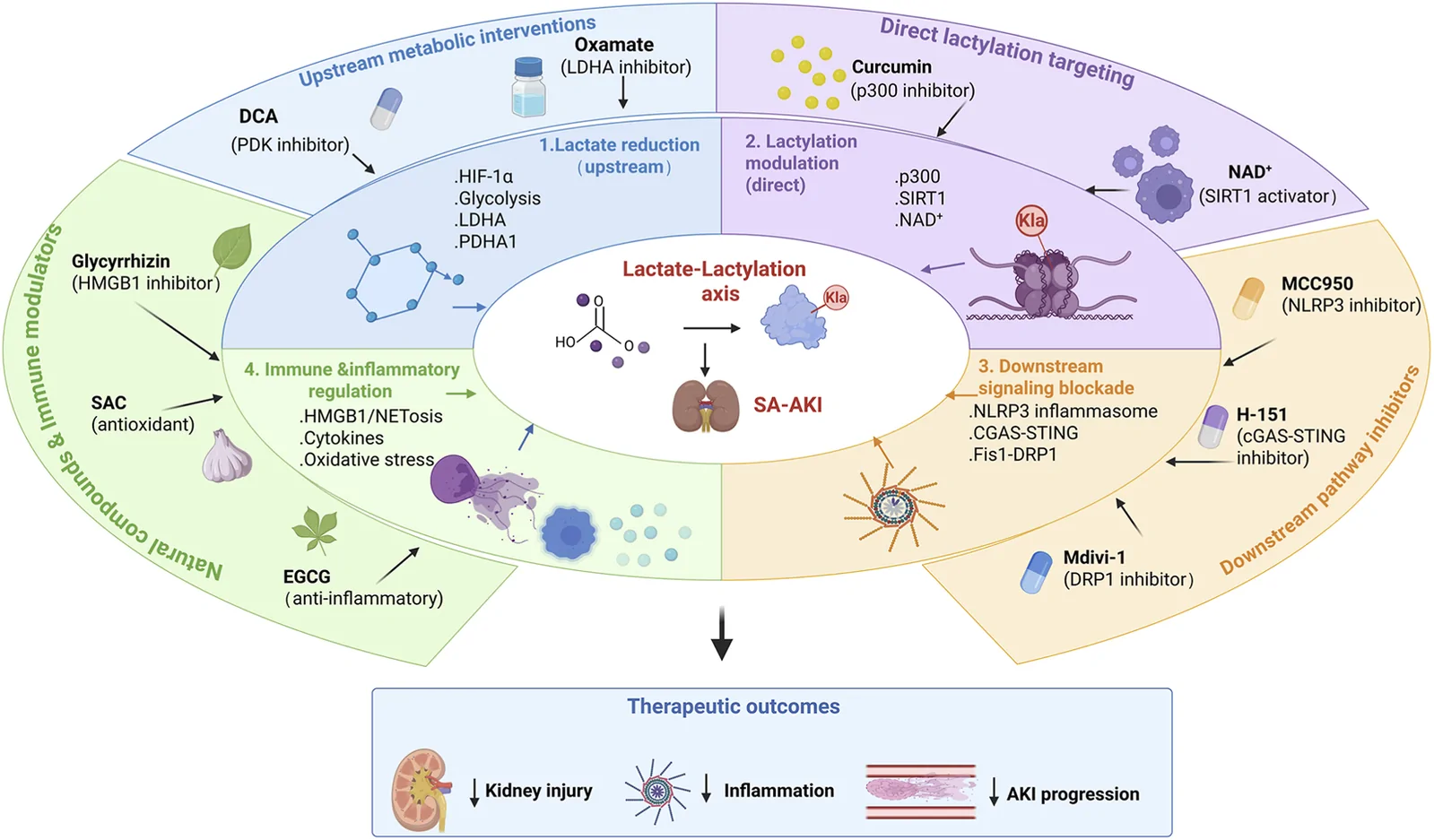
Therapeutic strategies targeting lactate metabolism, lactylation enzymes, and downstream injury pathways in SA-AKI.

Therapeutic translation also faces biological barriers. Lactylation participates in host defense, immune resolution, tissue repair, and possibly metabolic adaptation (Zhang et al., 2019; Hoque et al., 2014; Er et al., 2016; Yang et al., 2020; Zhang et al., 2021). Systemic lactylation inhibition could therefore impair protective immune responses or injury tolerance. Kidney-targeted delivery, cell-type-specific modulation, and time-windowed intervention will likely be necessary (Figure 3; Table 3).

**TABLE 3:** Therapeutic Strategies Targeting lactate-lactylation biology: evidence and limitations.

Targeted process	Intervention/example	Evidence/model	Rationale	Major limitation	References
Clinical lactate burden	Lactate-guided monitoring and lactate clearance	Clinical sepsis association	Risk assessment and dynamic response monitoring	Does not measure renal lactylation or causality	Ryoo et al. (2018); Zhang et al. (2025)
PDK/PDHA1 axis	Dichloroacetate (DCA)	Direct experimental SA-AKI	Restores pyruvate oxidation and reduces lactate/Fis1 K20la	No SA-AKI clinical validation; cell-context dependence	An et al. (2023)
LDH activity	GSK2837808A, oxamate	Experimental SA-AKI/lactate-induced AKI	Reduces lactate availability in selected models	Proximal-tubule LDHA can be protective in cisplatin AKI	An et al. (2023), Zhu et al., 2024; Lu et al. (2026)
Mitochondrial pyruvate entry	MPC modulation	Indirect rhabdomyolysis AKI	Reduced pyruvate uptake may induce protective redox adaptation	Not tested in SA-AKI; may conflict with DCA strategy	Rauckhorst et al. (2023), Rauckhorst et al. (2024)
p300-mediated lactylation	Curcumin; A-485/C646 concept	SA-AKI animal model for curcumin; general p300 biology	Reduces p300 expression/protein lactylation and inflammation	Bioavailability and specificity limitations	Zhang et al. (2019), Luo et al. (2025)
SIRT1/SPHK1 feedback	NAD + supplementation; PF-543	Direct experimental SA-AKI	Enhances delactylation and disrupts H3K18la/SPHK1/SIRT1 loop	Optimal timing and safety unknown	Huang et al. (2026)
Mitochondrial fission	Mdivi-1	Direct experimental SA-AKI	Limits Fis1/DRP1-dependent mitochondrial fragmentation	Downstream and not lactylation-specific	An et al. (2023)
Endothelial ROCK signaling	Y-27632	Direct experimental SA-AKI	Protects Ezrin/RhoA/ROCK-mediated endothelial barrier injury	Experimental; systemic vascular effects possible	Qiao et al. (2024)
Inflammasome activation	MCC950	Direct experimental SA-AKI downstream evidence	Limits NLRP3-mediated pyroptosis	Not lactylation-specific; translation uncertain	Huang et al. (2026)
cGAS-STING activation	H-151	Experimental SA-AKI pathway evidence	Reduces LDHB K156la-associated innate immune activation	Early-stage evidence	Luo et al. (2026)
HMGB1-NET axis	Glycyrrhizin; HMGB1 siRNA	Lactate-induced AKI/SA-AKI models	Reduces HMGB1 lactylation-related NET formation	Specificity and delivery issues	Zhu et al. (2024)

Therapeutic claims are intentionally conservative. Most strategies are preclinical, indirect, or downstream of lactylation rather than direct clinical anti-lactylation therapies.

Evidence note: proposed interventions range from direct SA-AKI animal evidence to indirect renal disease models; detailed evidence levels and limitations are listed in Table 3.

## 7 Conclusions and future perspectives

Lactate and lactylation form a biologically important but context-dependent regulatory network in SA-AKI. Direct SA-AKI studies support roles for H3K18la, Fis1 K20la, LDHB K156la, Ezrin K263la, HMGB1 lactylation, and ALDH2 K68la in mitochondrial injury, tubular cell death, endothelial barrier dysfunction, innate immune activation, and NET formation (An et al., 2023; Zhang et al., 2025; Huang et al., 2026; Qiao et al., 2024; Luo et al., 2026; Luo et al., 2025; Zhu et al., 2024; Wei et al., 2025; Huang et al., 2025; Li et al., 2025). However, lactate accumulation, lactic acidosis, transport, oxidation, metabolic routing, and lysine lactylation are distinct processes. A balanced model must recognize that lactate can be adaptive as a fuel, carbon source, redox-linked metabolite, and anti-inflammatory signal, while persistent or compartment-specific lactate availability may promote maladaptive lactylation.

Several priorities follow. First, SA-AKI studies should map cell-type-specific lactylation landscapes and distinguish renal parenchymal events from systemic immune-cell and endothelial mechanisms. Second, direct lactylation assays should be paired with lactate flux, transporter expression, acid-base status, and pyruvate-routing measurements. Third, therapeutic studies should avoid global assumptions about LDHA inhibition or pyruvate oxidation and instead define the injury context, cell type, and intervention window. Finally, lactylation-based biomarkers require prospective validation before clinical use. With these safeguards, lactate-lactylation biology may provide a useful framework for understanding SA-AKI heterogeneity and for identifying more precise therapeutic targets.
